# Characteristic Features of COVID-19 Illness Among Healthcare Workers: A Retrospective Analysis

**DOI:** 10.7759/cureus.18816

**Published:** 2021-10-16

**Authors:** Katherine McFarlin, Siji S Thomas, Terrance Kane, Josh Buell, Nita Thomas, Abhijit Shivkumar

**Affiliations:** 1 Internal Medicine, St. Bernards Medical Center, Jonesboro, USA

**Keywords:** covid-19 illness, healthcare workers, symptomatology, testing habits, gender comparison

## Abstract

Background

Frontline workers, who practice in a variety of settings, have been affected profoundly by the coronavirus disease 2019 (COVID-19) pandemic both professionally and personally. Due to the nature of their job responsibilities, many healthcare workers were exposed to a variety of settings to COVID-19. Because of its high transmissibility, testing of these individuals became prudent to limit the spread, particularly in healthcare settings, to avoid staffing issues as well as iatrogenic infections in patients. This study aimed to report symptoms and testing habits of healthcare workers (HCWs) who were tested positive for severe acute respiratory syndrome coronavirus 2 (SARS-CoV-2) illness.

Methods

At the beginning of each shift upon entering the hospital premises, all HCWs were screened for fever using thermal scanners. Also, they were interviewed about exposure history and other symptoms with a questionnaire. Those who experienced symptoms and presented to the employee health clinic for SARS-Cov-2 testing were asked to complete a questionnaire before testing regarding their symptomatology. Of nearly 1000 HCWs tested, 93 of them were positive for the COVID-19. Questionnaire data were then analyzed to identify the most and least common symptoms. Subgroup differences were also examined between the time of symptom onset and the date of the initial test.

Results

The most common reported symptoms were cough (81%), myalgia (75%), and headache (75%). An equal number of patients presented with myalgia and headache (75%). The mean number of days from the onset of symptoms to the day of testing was approximately 2.6 days; it was different for males (1.82 days) and females (2.8 days), although the results were not statistically significant. Only 53% of the participants experienced fever. The least reported symptoms were chest pain (18%) and rhinorrhea (9%). Infected workers were mainly those working in the COVID-19 unit or had a history of COVID-19 exposure while performing clinical duties.

Conclusions

Cough, myalgia, and headache were the most commonly reported symptoms. The least common reported symptoms were chest pain and rhinorrhea. Only 53% exhibited fever. Hence thermal scanning for fever detection may not be the ideal way to screen HCW for COVID-19 illness. The time from symptom onset to initial test didnot differ between female and male HCWs.

## Introduction

Since the outbreak of the coronavirus disease 2019 (COVID-19) in Wuhan, Hubei Province, China, in late December 2019, as of September 2021, there have been 223 million confirmed cases, including 4.6 million deaths globally [1]. This makes the current COVID-19 pandemic, the largest pandemic in the 21st century and the largest ever pandemic caused by one of the coronaviruses [2]. This pandemic has caused an unprecedented physical and emotional challenge to frontline healthcare workers (HCW). HCW are at increased risk for exposure than the general public. In fact, many frontline workers have lost their lives in the battle against this pandemic. As per World Health Organization in some countries, one in 10 health workers is infected with coronavirus [3]. However, only a few studies explored the demographics and symptomatology of COVID-19 among HCW. This study aimed to investigate the demographics, symptom pattern, and time lag between the time of testing and that of onset of symptoms among frontline HCW. This study may help identify patterns that will aid in reducing transmission among HCWs and mitigating community spread. Additionally, study findings may give an indirect assessment of the currently implemented protective measures in the healthcare setting including the need for thermal scan prior to start of shift work.

## Materials and methods

Institutional review board approval was obtained prior to conducting this study. Protocols and methods were established to protect healthcare information and maintain the confidentiality of participants. This retrospective observational study was conducted between July 2020 and October 2020 among HCW employed in a tertiary care center in Northeast Arkansas. All HCW above 18 years old who visited the employee health clinic for COVID-19 testing were given a questionnaire to describe their symptoms and subsequently underwent nasopharyngeal swab for COVID-19 testing using the RT-PCR technology. Of 983 HCW, 93 (9.4%) tested positive for the severe acute respiratory syndrome coronavirus 2 (SARS-CoV-2) virus. The questionnaire contained sociodemographic data and a list of 12 symptoms commonly seen in patients with COVID-19. The most and least common symptoms were identified by descriptive statistics. Additionally, the characteristics of testing time delay between males and females were compared by two-sample* t*-test.

## Results

**Figure 1:**
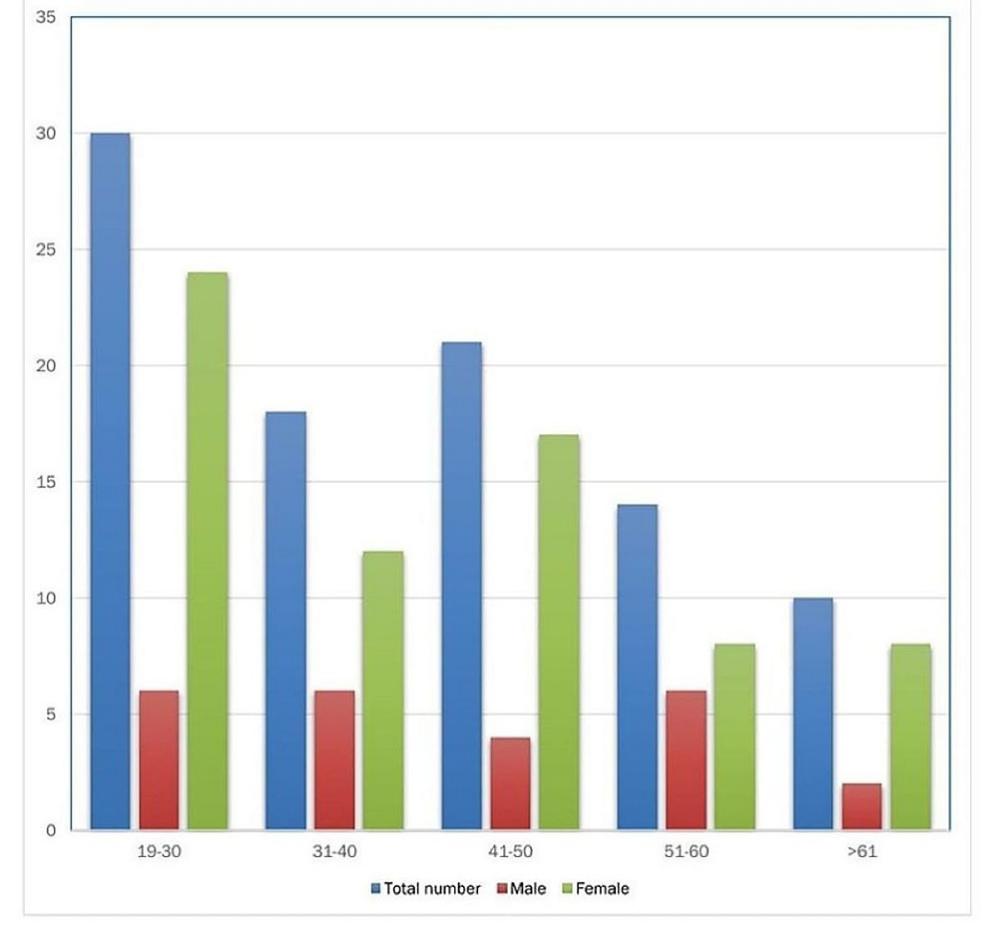
Population demographics

Of 983 HCW, 93 (9.4%) tested positive for the SARS-CoV-2 virus. These HCWs reported working predominantly in the covid isolation unit or had exposure at work. These HCWs were grouped according to their age. Data were collected for ages above 19 years to age over 60 years, employed in a healthcare facility in Northeast Arkansas. All age groups exhibited a higher female to male ratio as reported in Figure 1.

In the older age group, major symptoms included cough (100%), fatigue (75%), myalgia (69%), and headache (74%). There were differences in symptoms between those who were under and over 40 years old. Loss of smell and taste was primarily observed in patients over the age of 60 years. All patients above 40 years of age experienced cough. Interestingly, only 39% of patients between age 31 years and 40 years reported fever, and no employees over the age of 60 years reported fever as a presenting symptom. A higher proportion of patients across all age groups experienced headache as seen in Table 1.

**Table 1:** Symptoms reported by subgroup

Symptoms	19-30 years N(%)	31-40 years N(%)	41-50 years N(%)	51-60 years N(%)	>61 years N(%)
Fever	20(64)	7(39)	13(65)	9(64)	None
Cough	20(31)	11(61)	20(100)	14(100)	10(100)
Rhinorrhea	2(6)	1(5)	1(10)	1(7)	3(30)
Dyspnea	9(29)	3(17)	8(40)	4(29)	2(20)
Chest pain	6(19)	4(22)	4(20)	3(14)	None
Hypertension	1(3)	None	1(5)	1(7)	None
Tachycardia	6(19)	2(11)	3(15)	1(7)	None
Myalgia	26(84)	10(56)	15(75)	14(100)	5(50)
Fatigue	23(74)	8(44)	15(75)	6(43)	9(90)
Loss of smell/taste	14(45)	8(44)	7(35)	3(21)	8(80)
Headache	24(77)	13(72)	16(80)	10(71)	7(70)
Sore throat	17(55)	6(33)	7(35)	5(36)	4(40)
Nausea/vomiting	10(32)	6(33)	2(10)	5(36)	5(50)
Diarrhea	7(23)	1(5)	3(15)	6(43)	3(30)

**Figure 2:**
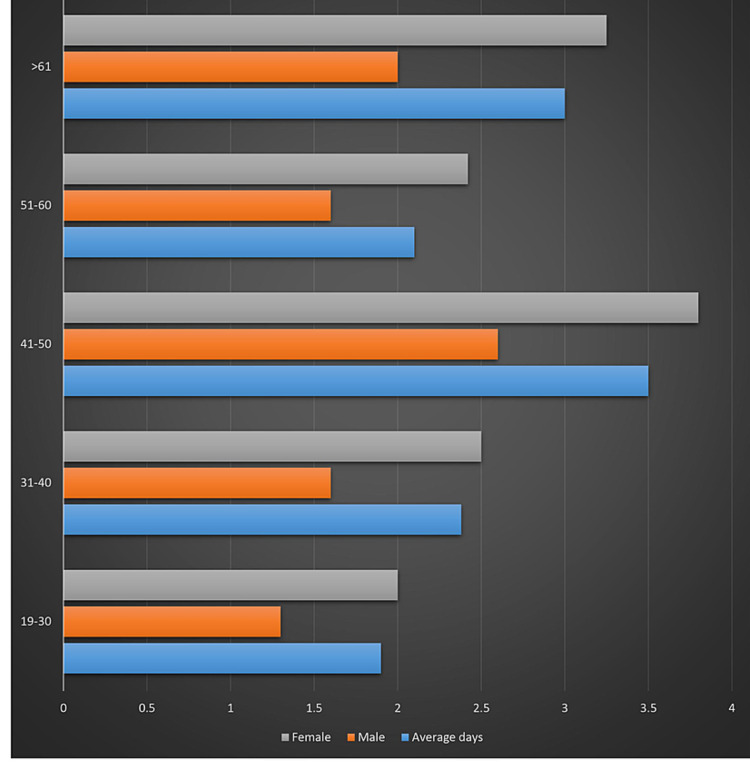
Mean delay time between symptom onset and initial test

The average days to testing from symptom onset was 2.56 days as seen in Figure 2 and Table 2. Furthermore, the average time between symptom onset and testing differed between males and females.

**Table 2:** Time between symptom onset and initial test by subgroup

Age group	Average days to test from symptom onset	Male	Female
19-30 years	1.9	1.3	2
31-40 years	2.38	1.6	2.5
41-50 years	3.5	2.6	3.8
51-60 years	2.1	1.6	2.42
>61 years	3	2	3.25
Total	Mean: 2.576 (SD:0.66)	Mean: 1.82, (SD: 0.50)	Mean: 2.8 (SD: 0.72)

## Discussion

Despite the overwhelming impact of COVID-19, the epidemiology of this viral disease in HCWs remains poorly explored. Occupational exposure has been cited as the major cause of illness. In a study done by Alshamrani et al., more males tested positive for SARS-CoV-2 virus compared to females [4]. In one CDC study involving 1423 HCWs that contracted COVID-19 and reported contact with a patient with laboratory-confirmed COVID-19, only 55% reported having such contact within 14 days before their illness. In addition, 92% reported having at least one of the following symptoms: fever, cough, and dyspnea. Two-thirds reported myalgia, and 65% reported headache [5]. In Washington, the most common initial symptoms were cough, fever, and myalgia. The median time from illness onset to symptoms that were currently used to screen for COVID-19 was two days. If myalgia and chills were included in the screening criteria at illness onset, case detection increased from 83% to 90%. Among those who were interviewed, approximately 65% reported working at a median of two days while experiencing symptoms [6].

In another study, HCW with COVID-19 were more likely to have an identified COVID-19 exposure, present less severely ill, and be less likely to be hospitalized than adult non-HCW with COVID-19 presenting to the emergency department. COVID-19 was also more frequent in females and those in the age group of 16-44 years [7]. In May 2020 in Wuhan, China, Lai et al. investigated the clinical characteristics and infection risk and found that the five most common symptoms were fever, fatigue, cough, sore throat, and myalgia. Contact with indexed patients and colleagues with infection, as well as community-acquired infection, were the main routes of exposure for HCWs. Most infections among HCW occurred during the early stage of disease outbreak. In addition, the infection rate was higher in non-frontline HCW than in frontline HCWs. This is a different observation compared with previous viral disease epidemics [8]. In another large study conducted by Lang et al. in the United Kingdom and the United States, the median age of HCWs infected with COVID-19 was 44 years. Compared with the general community, frontline HCW were more frequently female, had a slightly higher obesity prevalence, were more commonly smokers, and were also more likely to use nonsteroidal anti-inflammatory drugs. Moreover, 20% of the frontline HCWs reported at least one symptom associated with SARS-CoV-2 infection compared with 14% of the general population. Fatigue, loss of smell or taste, and hoarseness were especially frequent [9].

In this study, the most commonly reported symptoms were cough, fatigue, myalgia, and headache. Myalgia and headache were the most common among HCW aged below 40 years, and cough and headache among those aged over 40 years. Meanwhile, only 53% experienced fever. Conversely, the least reported symptoms were chest pain and rhinorrhea. All HCW above 40 years of age reportedly had cough, with an increasing incidence. In all age groups, male HCW tended to test earlier than female HCW. Additionally, the mean number of days from the onset of symptoms to the day of testing was 2.6 days. The proportion of HCW who tested positive for COVID-19 was similar to the meta-analysis findings done by Sahu et al. [10]. 

## Conclusions

This study explored the symptomatology and time lag between symptom onset and testing of COVID-19 among HCW in a tertiary care center in Northeast Arkansas. To our knowledge, this study is the first attempt to understand the difference in symptomatology and testing patterns according to age and sex among HCWs. The symptoms were considerably different between younger and older HCWs. The symptoms and testing time also differed according to sex. Considering that only 53% experienced fever, thermal scan use in hospitals and in other public utility areas for COVID-19 screening may have to be readdressed. Moreover, validated questionnaire, clinical scoring tools to predict pretest probability, and contact tracing may yield better results to identify source and transmission of highly contagious diseases among frontline HCW.
